# Supplementary material for: Usefulness of the cytokines expression of Th1/Th2/Th17 and urinary CD80 excretion in adult-onset minimal change disease
Source: PeerJ. 2020 Sep 8;8:e9854. doi: 10.7717/peerj.9854 (PMC7485503; doi:10.7717/peerj.9854)
Supplement: Supplemental Information 1 [file peerj-08-9854-s001.zip › raw data/cytokines antibody array/Figure 1.pptx]

## Slide 1
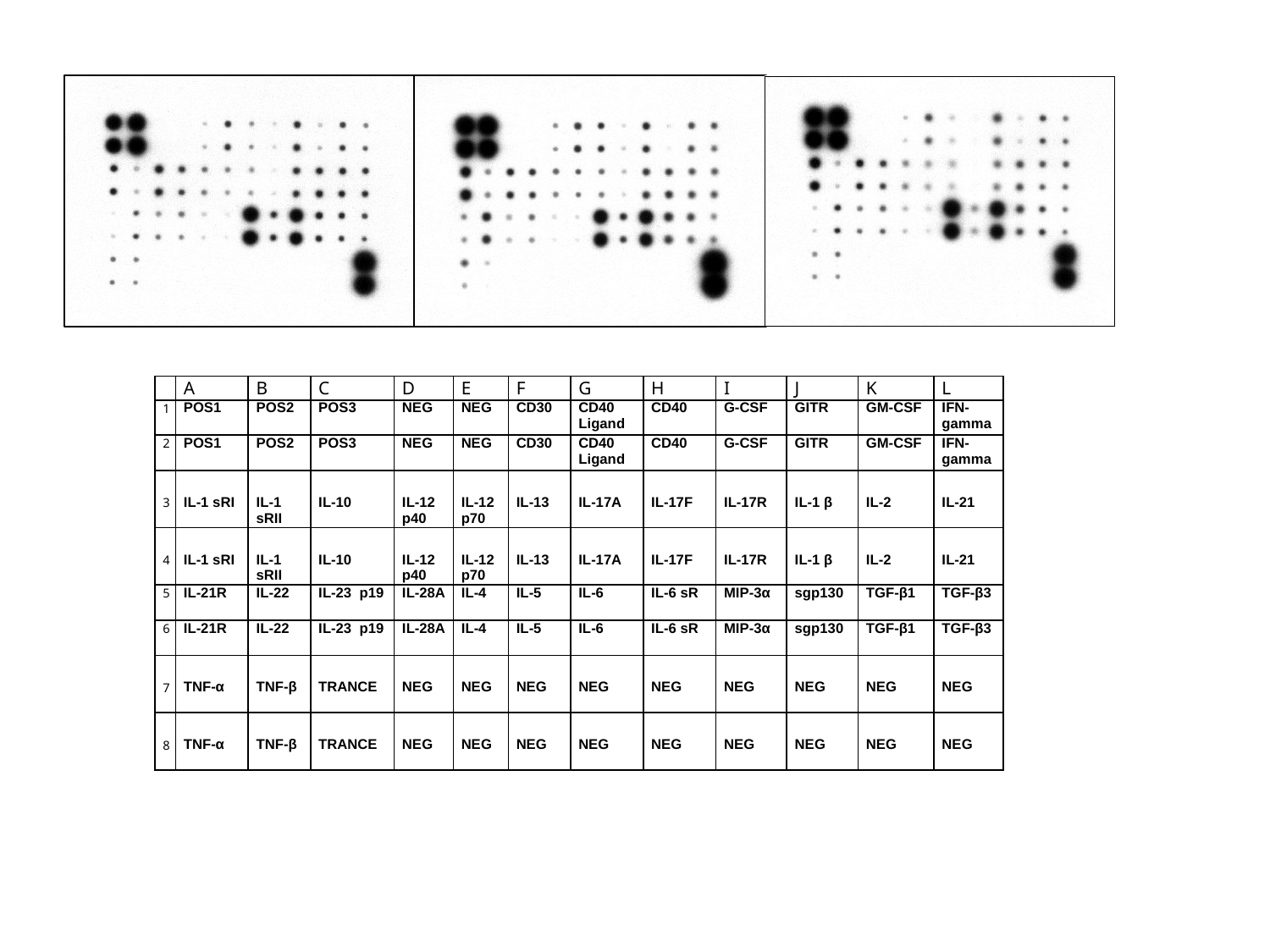

| | A | B | C | D | E | F | G | H | I | J | K | L |
| --- | --- | --- | --- | --- | --- | --- | --- | --- | --- | --- | --- | --- |
| 1 | POS1 | POS2 | POS3 | NEG | NEG | CD30 | CD40 Ligand | CD40 | G-CSF | GITR | GM-CSF | IFN- gamma |
| 2 | POS1 | POS2 | POS3 | NEG | NEG | CD30 | CD40 Ligand | CD40 | G-CSF | GITR | GM-CSF | IFN- gamma |
| 3 | IL-1 sRI | IL-1 sRII | IL-10 | IL-12 p40 | IL-12 p70 | IL-13 | IL-17A | IL-17F | IL-17R | IL-1 β | IL-2 | IL-21 |
| 4 | IL-1 sRI | IL-1 sRII | IL-10 | IL-12 p40 | IL-12 p70 | IL-13 | IL-17A | IL-17F | IL-17R | IL-1 β | IL-2 | IL-21 |
| 5 | IL-21R | IL-22 | IL-23 p19 | IL-28A | IL-4 | IL-5 | IL-6 | IL-6 sR | MIP-3α | sgp130 | TGF-β1 | TGF-β3 |
| 6 | IL-21R | IL-22 | IL-23 p19 | IL-28A | IL-4 | IL-5 | IL-6 | IL-6 sR | MIP-3α | sgp130 | TGF-β1 | TGF-β3 |
| 7 | TNF-α | TNF-β | TRANCE | NEG | NEG | NEG | NEG | NEG | NEG | NEG | NEG | NEG |
| 8 | TNF-α | TNF-β | TRANCE | NEG | NEG | NEG | NEG | NEG | NEG | NEG | NEG | NEG |

## Slide 2
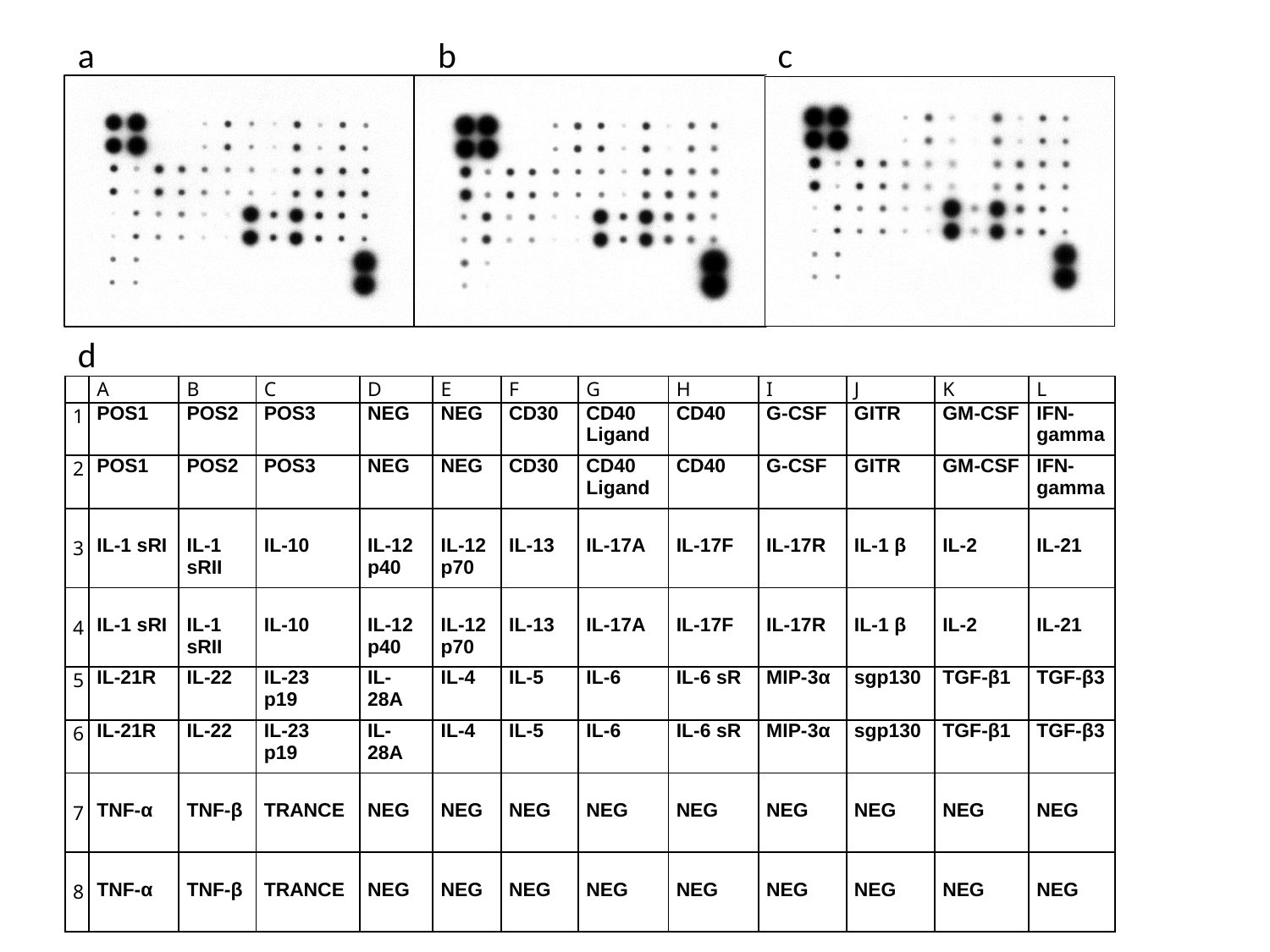

a
b
c
d
| | A | B | C | D | E | F | G | H | I | J | K | L |
| --- | --- | --- | --- | --- | --- | --- | --- | --- | --- | --- | --- | --- |
| 1 | POS1 | POS2 | POS3 | NEG | NEG | CD30 | CD40 Ligand | CD40 | G-CSF | GITR | GM-CSF | IFN- gamma |
| 2 | POS1 | POS2 | POS3 | NEG | NEG | CD30 | CD40 Ligand | CD40 | G-CSF | GITR | GM-CSF | IFN- gamma |
| 3 | IL-1 sRI | IL-1 sRII | IL-10 | IL-12 p40 | IL-12 p70 | IL-13 | IL-17A | IL-17F | IL-17R | IL-1 β | IL-2 | IL-21 |
| 4 | IL-1 sRI | IL-1 sRII | IL-10 | IL-12 p40 | IL-12 p70 | IL-13 | IL-17A | IL-17F | IL-17R | IL-1 β | IL-2 | IL-21 |
| 5 | IL-21R | IL-22 | IL-23 p19 | IL-28A | IL-4 | IL-5 | IL-6 | IL-6 sR | MIP-3α | sgp130 | TGF-β1 | TGF-β3 |
| 6 | IL-21R | IL-22 | IL-23 p19 | IL-28A | IL-4 | IL-5 | IL-6 | IL-6 sR | MIP-3α | sgp130 | TGF-β1 | TGF-β3 |
| 7 | TNF-α | TNF-β | TRANCE | NEG | NEG | NEG | NEG | NEG | NEG | NEG | NEG | NEG |
| 8 | TNF-α | TNF-β | TRANCE | NEG | NEG | NEG | NEG | NEG | NEG | NEG | NEG | NEG |
