# Supplementary figures and images for: Usefulness of the cytokines expression of Th1/Th2/Th17 and urinary CD80 excretion in adult-onset minimal change disease
Source: PeerJ. 2020 Sep 8;8:e9854. doi: 10.7717/peerj.9854 (PMC7485503; doi:10.7717/peerj.9854)

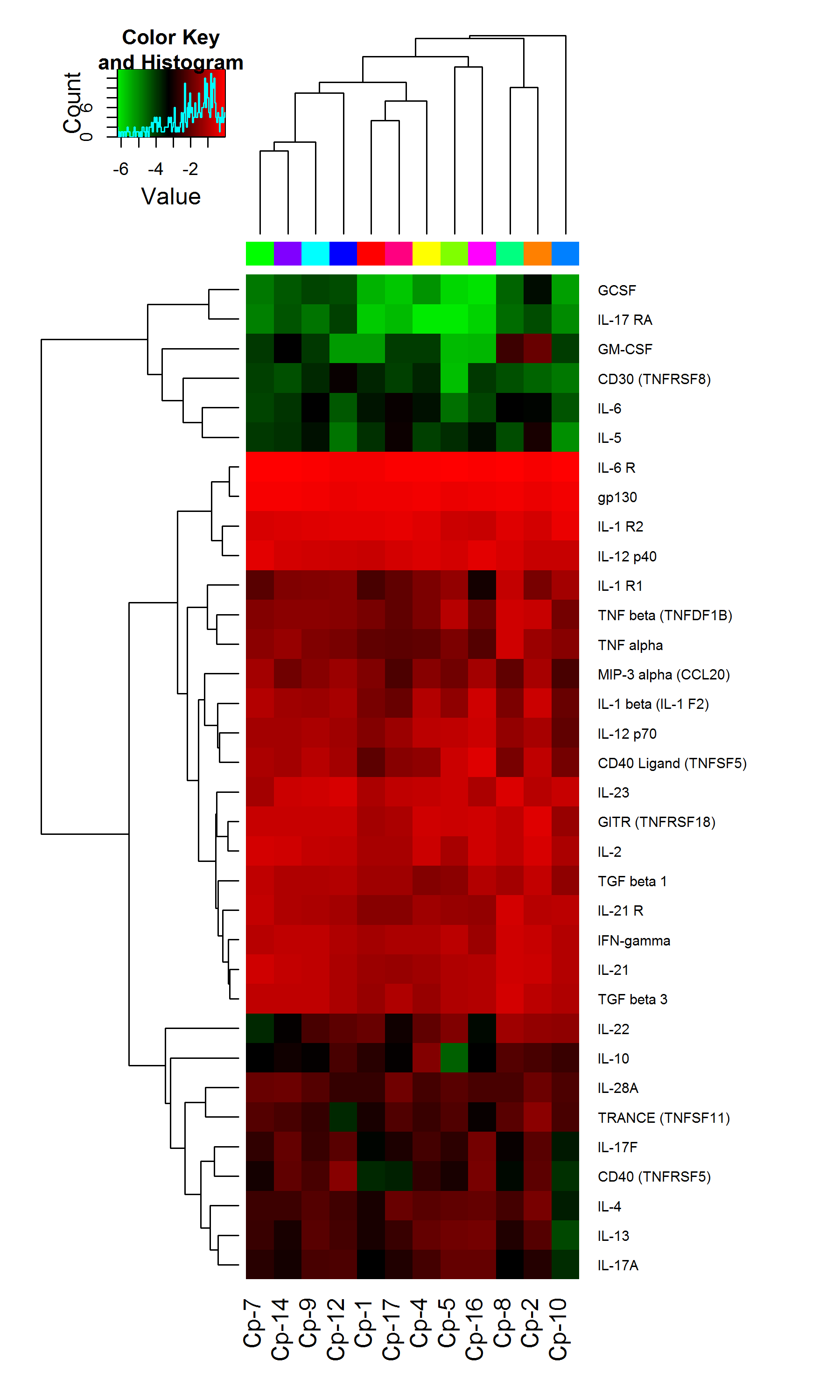

Supplement: Supplemental Information 1 [file peerj-08-9854-s001.zip › raw data/cytokines antibody array/cluster.png]
